# Exploring the psychological well-being of midwives after maternal deaths: an integrative review

**DOI:** 10.3389/fpsyg.2026.1676481

**Published:** 2026-03-26

**Authors:** Nkhensani Florence Mabunda, Mashudu Tulelo

**Affiliations:** Nursing Department, Sefako Makgatho Health Sciences University, Pretoria, Gauteng, South Africa

**Keywords:** coping mechanisms, maternal mortality, mental health, midwives, psychological

## Abstract

**Introduction:**

The mental health of midwives is crucial both for their professional performance and personal well-being, particularly following traumatic incidents like maternal fatalities. Though these experiences can be emotionally draining, research is scarce on the coping mechanisms employed by midwives in such situations. This integrative review seeks to investigate and combine current studies concerning the mental health of midwives after experiencing maternal mortality, pinpointing the psychological effects, coping strategies, and available support networks.

**Methods:**

An integrative review was carried out by searching databases including PubMed, PsycINFO, CINAHL and Google Scholar. Studies from 2015 to 2025 were selected if they examined midwives’ psychological reactions to maternal deaths. Information was gathered, assessed, and organized around main themes.

**Results:**

Ten studies were examined in the review, uncovering several psychological effects on midwives such as guilt, anxiety, depression, and burnout. Coping mechanisms encompassed peer support, professional counseling, and debriefing sessions. Moreover, the level of institutional support demonstrated significant variation.

**Discussion:**

This integrative review outlines the considerable psychological distress experienced by midwives following maternal deaths, such as feelings of grief, guilt, anxiety, and secondary trauma, particularly in settings with limited resources. Coping mechanisms like peer support, debriefing sessions, and spiritual practices provide some relief, yet frequently prove insufficient due to a lack of proper institutional backing. The review advocates for further research into the long-term effects, strategies to enhance resilience, and the formulation of trauma-informed policies and mental health services to better support midwives in a variety of healthcare contexts.

## 1 Introduction

The psychological well-being of midwives is a global concern in the context of providing maternal health services that decrease productivity in the workplace (Shaghaghi et al., 2019). The escalating concern pertains to the insufficiency of resources imperative for maternity services, which significantly contributes to maternal mortality. This includes deficits in both human resources and equipment. The literature indicates that the occurrence of maternal fatalities has a detrimental impact on midwives, who frequently endure emotional distress and face difficulties in executing their professional responsibilities (Muliira and Bezuidenhout, 2015). Compounding this concern is a significant challenge in the midwifery profession in Australia (Sheehy and Baird, 2022), which contributes to workforce attrition and retention issues due to psychological stress, exhaustion, and job dissatisfaction (Wahlberg et al., 2020). Previous research has shown that witnessing maternal death negatively affects midwives’ psychology, cognitive alterations, quality of work life and even family life (Çankaya and Dikmen, 2020; Dartey and Phuma-Ngaiyaye, 2020).

As noted by Aydın and Aktaş (2021), midwives who experience maternal deaths frequently suffer deep psychological effects, sometimes destabilizing them emotionally and leading them to contemplate leaving the profession. This is similar to Cleveland et al. (2020) who support these findings, showing that midwives are especially susceptible to posttraumatic stress symptoms due to their encounters with maternal death events. Such stress responses not only impact their mental health but also jeopardize their overall quality of life. Reports often include emotional reactions like persistent intrusive thoughts, anxiety, depression, and helplessness, significantly affecting both personal well-being and job performance. In line with this, another study confirmed that the psychological well-being of midwives is profoundly affected by witnessing maternal deaths, influencing their professional behavior (Nieuwenhuijze et al., 2024). Additionally, research into the prevalence of post-traumatic stress disorder (PTSD) among Italian midwives who have observed maternal deaths identified a high risk of developing work-related PTSD (Guzzon et al., 2024). This heightened risk is linked to the emotionally intense and often sudden nature of maternal deaths, which leave enduring psychological effects. These studies collectively highlight the critical need for targeted mental health support and institutional structures to safeguard midwives’ well-being and promote safe, effective maternal care.

Several studies have emphasized that both personal and professional life gain meaning when individuals effectively handle challenges faced at work, while Meeusen et al. (2024) highlights that managing work-related stress is essential for achieving and sustaining occupational well-being. A study conducted in Ghana on midwives’ coping challenges post-maternal deaths revealed that many found it difficult to accept patient losses, often leading to occupational trauma and emotional turmoil (Dartey et al., 2025; Minooee et al., 2020). Moreover, a scoping review on the broader effects of birth trauma on midwives identified several factors contributing to psychological distress. These factors include risk sensitivity, understanding of legal and ethical responsibilities, clinical expertise, confidence in decision-making, and previous traumatic experiences (Minooee et al., 2020; Bingham et al., 2023). The findings highlight the critical need for workplace measures that help midwives cope with traumatic events to protect their mental health and improve maternal care quality.

As a result, the midwifery experts recommend interventions to provide organizational support, execute self-care, and integrate mental healthcare into maternity services as strategies to improve the psychological wellbeing of midwives (Moran et al., 2023). What is known about midwives’ psychological well-being is that they experience shock, fear, depression, stress, and insomnia that lead to psychological trauma and emotional exhaustion after witnessing maternal deaths (Bingham et al., 2023; Doherty and O’Brien, 2023; Bramble, 2024). Based on the above literature, the authors decided to conduct the study to extend the literature to develop a comprehensive understanding of the topic from a combination of all forms of available evidence (Campbell et al., 2023; Mohale and Nyangu, 2023; Souza et al., 2024; Smith et al., 2025). Therefore, this paper aims to provide an integrated review of assimilated research data from various research designs to reach conclusions that are comprehensive and reliable on the psychological well-being of midwives after maternal deaths.

Although midwives play a crucial role in providing care and support during childbirth, the emotional toll they experience after a maternal death is largely unacknowledged in the existing literature. Research has indicated that healthcare professionals involved in traumatic events like maternal death may suffer from emotional exhaustion and secondary trauma (Mendelson, 2024; Segal and Kagan, 2025), but the results remain inconsistent. The preponderance of extant literature has predominantly concentrated on midwives, who, in particular, encounter distinctive challenges due to their direct engagement in labor and delivery processes, alongside their emotional and professional commitment to maternal and neonatal outcomes (Ernawati, 2024), leaving the lack of support systems for midwives, along with insufficient mental health resources, exacerbates the psychological risks they face after such traumatic experiences unexplored (Taylor et al., 2024).

Although recent studies have highlighted the importance of professional debriefing and psychological support in mitigating burnout and trauma among healthcare providers (Baum et al., 2025), such practices have not been extensively tailored or researched for midwives. Given the paucity of focused research, there is a need for an integrative review that synthesizes existing findings on the psychological well-being of midwives after maternal deaths, identifies gaps in the literature, and provides evidence-based recommendations for supporting midwives’ mental health. This integrative review is framed by Ben-Zur’s Transactional Model of Stress and Coping (Ben-Zur, 2020), builds upon the foundational work of Lazarus and Folkman (1987). By concentrating on Ben-Zur’s theoretical model, this review employs a cohesive theoretical framework to elucidate the psychological implications of maternal death on midwives, as well as their experiential responses. The model delineates stress as a dynamic interaction between the individual and environmental factors, whereby midwives perceive maternal death as a potential threat, harm, or challenge and assess their available coping resources. Furthermore, the model distinguishes between problem-focused coping strategies—such as debriefing, clinical supervision, and problem-solving—and emotion-focused strategies, which include seeking emotional support, avoidance, or participation in spiritual activities.

The present framework facilitates the integrative review by offering a systematic methodology for the analysis and synthesis of literature concerning the psychological experiences of midwives. It enables the classification of emotional outcomes—such as anxiety, guilt, grief, and trauma-based on the effectiveness of coping mechanisms and underscores both internal resources (e.g., resilience, self-awareness) and external resources (e.g., institutional and peer support) that impact psychological well-being. Employing Ben-Zur’s model as the foundational framework augments theoretical coherence, informs organizational interventions, and integrates psychological theory with evidence-based practice to enhance midwives’ coping strategies and emotional resilience in the aftermath of maternal death.

## 2 Materials and methods

### 2.1 Design

An integrative review was chosen for this study because it allows for the inclusion of diverse methodologies (qualitative, quantitative, and mixed methods) and offers a comprehensive view of the research on a topic. This integrative review aims to synthesize the existing literature on the psychological well-being of midwives following maternal deaths, exploring the impacts, coping strategies, and available support mechanisms, with an emphasis on identifying key factors that influence their mental health and well-being. By examining the existing literature from 2015 to 2025, this review will provide a comprehensive understanding of the psychological impact on midwives and offer guidance on how healthcare systems can better support these essential professionals.

### 2.2 Procedure

The researchers of this study followed the steps outlined in Whittemore and Knafl (2005): (1) problem identification, (2) literature search, (3) data evaluation, (4) data analysis, and (5) presentation of findings were employed to deliver an extensive insight into the subject (Bingham et al., 2023; Moran et al., 2023). A review was deemed suitable as it holds an important position in evidence-based practice for performing integrative reviews, ensuring a thorough and methodical approach (Doherty and O’Brien, 2023).

#### 2.2.1 Identification of the research problem

Given the rising need for midwifery services, it is evident that there is a lack of comprehension of the psychological effects of maternal death amongst midwives. Maternal mortality continues to pose a major public health challenge, particularly in low- and middle-income nations where the dangers associated with childbirth are magnified (Ekwuazi et al., 2023). There has been significant emphasis on the psychological consequences of maternal death for the families affected and for healthcare workers; however, the mental health of midwives working under these high-stress conditions has not been adequately examined. Midwives, who frequently witness these traumatic maternal deaths, are vulnerable to various forms of emotional and psychological distress, such as grief, burnout, anxiety, and depression. Despite their vital role in supporting childbirth, the emotional burden midwives endure when faced with maternal death is mostly overlooked in current studies. Evidence shows that healthcare workers involved in traumatic events like maternal death can experience emotional exhaustion and secondary trauma (Ernawati, 2024; Kendall-Tackett and Beck, 2022).

Midwives, in particular, encounter specific challenges due to their hands-on role in the birth process and their emotional and professional commitment to maternal and infant health outcomes (Mendelson, 2024). The absence of adequate support systems and mental health resources for midwives aggravates the psychological risks they face following such traumatic events (Segal and Kagan, 2025; Ernawati, 2024). Additionally, there is a notable lack of research on coping strategies, resilience, and organizational support for midwives confronting maternal death. Although existing studies acknowledge the value of professional debriefing and emotional support in reducing burnout and trauma in healthcare providers (Taylor et al., 2024; Evans et al., 2023), these interventions have not been extensively adapted or examined specifically for midwives. Due to the scarcity of targeted research, there is a pressing need for a comprehensive review that consolidates current findings on midwives’ psychological well-being after maternal deaths, identifies existing gaps, and offers evidence-based recommendations to support their mental health.

##### 2.2.1.1 Inclusion and exclusion criteria

The selection criteria, both inclusion and exclusion, for this integrative review were established to decide which articles from the literature could be incorporated into the study. Search filters were applied to include studies published between 2015 and 2025 in English-language journals. Previous studies were omitted to enhance the temporal efficiency of search procedures, while concurrently limiting the magnitude of generated research output. Studies that focus on midwives engaged in maternal death cases within clinical or hospital environments to explore the psychological health, emotional distress, psychological trauma, burnout, coping methods, support systems for professionals, and resilience in various healthcare environments, including hospitals, maternity clinics, and community health settings, were included.

#### 2.2.2 Literature search

Two reviewers used electronic databases for the literature search process of relevant literature with the consultation of a university librarian. Terms such as psychological well-being,” “midwives,” “maternal death,” “emotional impact,” “coping mechanisms” “healthcare professionals’ mental health.” A systematic search was performed using electronic databases including PubMed, CINAHL, PsycINFO, and Google Scholar, where two reviewers (NFM, MT) proceeded to independently screen on the three separate levels of titles, abstracts, and full texts based on the inclusion criteria. The search employed a blend of keywords alongside the Boolean operators ‘AND’ and ‘OR’ to integrate all concepts, aiming to gather pertinent articles.

These keywords were employed in multiple combinations to guarantee the incorporation of studies focusing on the psychological well-being of midwives following maternal deaths. A variety of keywords were used in different combinations to ensure the research captured the relevant studies. Specifically, the focus was on studies examining how midwives’ psychological well-being is affected after maternal deaths. It mentions specific studies by Moran et al. (2023) and Tzamakos et al. (2024), indicating that these studies were part of the body of research considered pertinent to the topic. The search strategy was narrowly tailored to include only English-language articles. This suggests a focus on easily accessible articles in English-language journals to maintain the relevance and applicability of the evidence gathered. Additionally, the process involved not just identifying relevant qualitative, quantitative, and mixed methods studies by their titles or abstracts but also included comprehensive readings of complete texts. This indicates a detailed approach to ensure that the studies are suitable and directly address the research question about midwives’ psychological well-being after experiencing maternal deaths. Overall, this approach illustrates a methodical and thorough search methodology to gather relevant academic literature on the specified topic.

**Figure 1:**
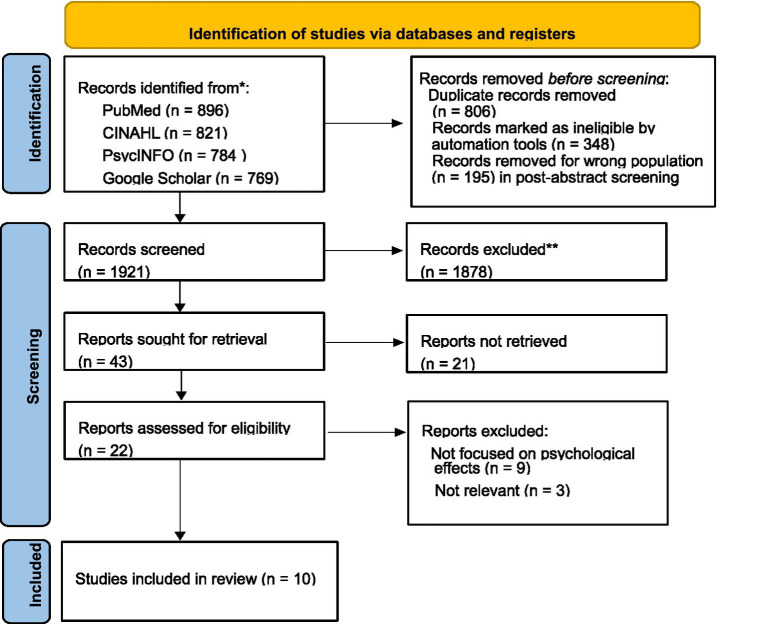
PRISMA flow diagram source: Adapted from: Page et al. (2021). *Consider, if feasible to do so, reporting the number of records identified from each database or register searched (rather than the total number across all databases/registers). **If automation tools were used, indicate how many records were excluded by a human and how many were excluded by automation tools.

Furthermore, selected articles were chosen by examining the titles and abstracts of pertinent studies, systematically excluding any that did not meet the inclusion criteria. In addition, the PRISMA flowchart by Page et al. (2021) was applied to broaden the scope of study screening for relevance and potential inclusion, as shown in Figure 1. These procedures took place under the guidance of seasoned researchers to maintain the rigor required for this integrative review. Moreover, excluded were all studies that either predate 2015 or are set after June 2025, those not published in English, or those lacking pertinent psychological outcomes. Additionally, studies concentrating on healthcare professionals other than midwives, like obstetricians and other medical practitioners, or not addressing maternal deaths, were also omitted.

Figure 1 shows a Preferred Reporting Items for Systematic Reviews and Meta-Analyses (PRISMA) flow diagram to summarize the key stages of study selection and inclusion/exclusion criteria. The PRISMA flow diagram is used to illustrate the stages of selecting studies for inclusion in a systematic review or meta-analysis. The flow diagram typically involves the following four stages: The process of searching various databases to gather potentially relevant studies. In this case, the databases searched included PubMed, CINAHL, PsycINFO, and Google Scholar, resulting in a total of 3,270 records (PubMed: 896, CINAHL: 821, PsycINFO: 784, and Google Scholar: 769). The initial filtering of the identified studies to exclude duplicates and obviously irrelevant records. From the 3,270 records identified, 1,349 were removed, potentially due to duplication or irrelevance to the study criteria. A more detailed assessment of the studies to see if they meet the predefined inclusion and exclusion criteria. In this integrative review, ambiguous screening categories arose during the title and abstract screening phases when studies partially fulfilled the inclusion criteria or had an unclear connection to the subject of midwives’ psychological experiences after maternal death. These studies were thoroughly re-assessed and deliberated among the reviewers until a consensus was achieved on whether to include or exclude them. The final set of studies that are deemed suitable for inclusion in the synthesis (systematic review or meta-analysis). The calculation “Total 3,270–1,349 = 1921″ represents the number of records remaining after the initial screening stage, showing that 1,921 studies were considered for the eligibility phase.

#### 2.2.3 Appraisal of study quality

Appraisal of study quality refers to the process of evaluating the overall quality and reliability of a study. This involves examining various aspects of the study, such as its design, data collection methods, analysis, and conclusions, to determine how well it meets certain standards or criteria. The appraisal helps in assessing the validity and relevance of the study’s findings. Appraisal of the study quality process and purpose of evaluating the quality of articles for this review was to ensure that the assessment was clear and transparent, which involved both researchers summarizing their findings. Each article selected for the review underwent a thorough quality check to uphold the overall integrity of the review. The ‘Critical Appraisal Skills Programme (CASP) checklist’ was the tool used for this evaluation process. This checklist helps assess the quality of studies by examining how well a study was conducted, how it communicates its findings, and the significance of its contributions. Any studies that did not meet the required quality standards set by this checklist were excluded from the review, ensuring that only high-quality research was included.

Table 1 presents the CASP checklist, a useful tool composed of various questions designed to assist researchers in assessing the rigor and applicability of scientific studies. This enables researchers to evaluate the quality and relevance of these studies (Humayoun et al., 2024). Each article was frequently rated by both researchers using a numeric scale (e.g., 1–5) and a quality level (e.g., “high,” “medium,” or “low”) based on the criteria listed in Table 1.

**Table 1:** Quality assessment of the articles.

Authors and year	Title	Relevance to research question	Data analysis quality (1–5)	Study design quality (1–5)	Overall quality rating	1st rating	2nd rating	Final rating
Bentil et al. (2025)	High	4	5	High	High	High	High	High
Segal and Kagan (2025)	Medium	4	3	Medium	Medium	Medium	Medium	Medium
Navon et al. (2025)	High	5	4	High	High	High	High	High
Qian et al. (2024)	Low	3	2	Low	Low	Low	Low	Low
Hanna et al. (2024)	High	4	5	High	High	High	High	High
Kave et al. (2023)	Medium	4	3	Medium	Medium	Medium	Medium	Medium
Garcia-Catena et al. (2023)	High	5	4	High	High	High	High	High
Dartey et al. (2019a)	Low	3	2	Low	Low	Low	Low	Low
Dartey et al. (2017)	High	4	5	High	High	High	High	High
Muliira and Bezuidenhout (2015)	Medium	4	3	Medium	Medium	Medium	Medium	Medium

This paper employs an integrative review approach to gain a comprehensive understanding of the research and consolidate findings from multiple healthcare sources (Long et al., 2020). This method commonly highlights knowledge gaps and guides future research directions, especially in disciplines such as nursing, where different methodologies explore intricate phenomena. Unlike systematic reviews, which concentrate on a limited selection of studies, an integrative review permits the inclusion of a broader array of study designs. Such reviews can integrate results from diverse research formats, including experimental, non-experimental, qualitative, and case studies that typically examine a narrower spectrum of study types (Dhollande et al., 2021).

#### 2.2.4 Data analysis and synthesis

Thematic analysis and synthesis were utilized to discover common patterns and themes related to midwives’ psychological experiences and coping mechanisms following maternal fatalities. Drawing on the work of Thomas and Harden (2008), the authors aimed to summarize the psychological outcomes and support systems accessible to midwives. By identifying these patterns and themes, they explored the psychological impacts and coping strategies used by midwives in such scenarios. A wide range of studies was reviewed to create a detailed summary of how these experiences influence midwives’ mental health and the support structures that exist for them. The goal was to gain a detailed understanding of the effects of these challenging events on midwives to potentially enhance support mechanisms. By amalgamating findings from different studies, the researchers intended to offer an in-depth insight into how these experiences affect midwives’ psychological well-being in these contexts, in addition, the ethical approval was unnecessary for this study as it is a comprehensive review of existing literature. Nonetheless, the studies incorporated in the review underwent critical evaluation concerning ethical aspects.

#### 2.2.5 A data extraction

A data extraction process that collects detailed and pertinent information from various studies included in a review. This systematic data extraction process aimed to ensure that the information was detailed, accurate, and relevant. This rigorous method allowed for the effective comparison and integration of findings from different sources, enhancing the overall analysis. The passage emphasises the importance of this detailed approach in ensuring that the review results are credible, valid, and trustworthy. It also notes that the final search of the database was completed in June 2025. For better understanding and clarity, a summary highlighting the key characteristics of the studies, such as their objective of the study, design and main findings, is provided in Table 2.

**Table 2:** Papers included in this integrative review.

Authors, year	Aim	Study design	Main findings
Bentil et al. (2025)	To investigate and portray the impact of maternal fatalities on midwives.	Descriptive qualitative design	Maternal fatalities and the accompanying audit procedures significantly impact the health of midwives, both physically, psychologically, and professionally. It is strongly recommended that systems be fortified to offer support to midwives dealing with maternal deaths.
Segal and Kagan (2025)	To explore the relationships between exposure to traumatic events, post-traumatic symptoms, and individual resilience with work-related quality of life and organizational commitment in hospital midwives.	Cross-sectional study	Repeated exposure to traumatic incidents generally elevates both the frequency and intensity of post-traumatic symptoms. This often leads to a rise in professional burnout and compassion fatigue, while compassion satisfaction tends to diminish. Conversely, greater compassion satisfaction and lower levels of professional burnout were associated with a stronger organizational commitment.
Navon et al. (2025)	To assess the frequency with which midwives experience traumatic births and to examine the link between their self-compassion and psychological well-being within the framework of these traumatic events.	Cross-sectional correlational study	Self-compassion and psychosocial well-being were observed to be moderately high. A significant interaction emerged between the primary effects of self-compassion and experiencing a traumatic birth within the last year on psychosocial well-being.
Qian et al. (2024)	To examine a proposed model detailing the particular influence pathways linking organizational support, confidence in perinatal bereavement care (PBC), secondary traumatic stress, and emotional exhaustion in nurses and midwives	A descriptive, cross-sectional survey	Organisational support positively and directly influenced confidence in PBC, while showing a significant, negative direct relationship with both secondary traumatic stress and emotional exhaustion. Confidence in PBC was found to exert a positive direct influence on secondary traumatic stress, along with a positive indirect influence on emotional exhaustion through secondary traumatic stress. Moreover, secondary traumatic stress had a notable, direct impact on emotional exhaustion.
Hanna et al. (2024)	To assess the impact of experienced emotional states on the risk of occupational burnout among midwives who have dealt with a maternal death.	Cross-sectional study	Depression considerably influences the likelihood of encountering occupational burnout, emphasizing the importance of creating and applying effective support strategies for midwives dealing with challenging emotions associated with patient deaths during their shifts.
Kave et al. (2023)	To explore the coping behaviors and support needs of midwives caring for women with perinatal loss.	Qualitative, exploratory, descriptive design	Regarding midwives’ strategies for dealing with perinatal loss, they emphasized the importance of receiving support from management alongside psychological and emotional assistance. Establishing psychological and emotional support systems tailored to the specific needs of each unit. Additionally, they expressed the necessity for redesigning the layout of the labor wards and highlighted that the issue of staff shortages requires immediate attention.
Garcia-Catena et al. (2023)	To outline the approaches employed by nurses and midwives in managing perinatal death experiences and sustaining job satisfaction.	Systematic literature review	Tactics for managing stress involve sharing emotions with colleagues, actively listening to the families served, undergoing training, and obtaining support from the institution.
Dartey et al. (2019a)	To describe the approaches employed by midwives to alleviate the impact of maternal fatalities and their methods of adjusting to their work settings.	Qualitative exploratory descriptive design	Informal Coping Strategies are recognized as methods employed by midwives to manage the effects of maternal deaths. These tactics encompass individualized non-professional help, peer support, assistance from family, self-care practices, spiritual support, and structured support through the review of maternal fatalities.
Dartey et al. (2017)	To investigate and detail midwives’ experiences of emotional distress following maternal death	Qualitative exploratory descriptive design	Midwives have been undergoing feelings of sadness, trauma, chaos, and exposure to death, all perceived as distressing, painful, and inhumane. Implementing support programs within hospitals was acknowledged as a beneficial strategy to help staff cope with these challenges, lessen the stress experienced by midwives, improve service quality, and boost job satisfaction.
Muliira and Bezuidenhout (2015)	To examine the psychological effects of being professionally exposed to maternal mortality and the strategies employed by midwives in rural settings to cope with such events	Quantitative descriptive design	Most midwives who observed maternal fatalities experienced moderate to high levels of death anxiety (93%), mild to moderate death obsession (71%), and mild death depression (53%). They generally managed their distress through strategies like active coping, venting, positive reframing, self-distraction, and planning.

Table 2 presents data systematically gathered from the chosen studies with the aid of a uniform data 337 extraction form. Essential details, such as the author(s), objective(s), research designs, and a synopsis 338 of the findings, were documented (Clipstone and Ambrosio, 2024) as shown in Table 2. This approach facilitated effective 339 comparison of the studies and guaranteed that significant information was collected for analysis 340 (Baker and Stewart, 2020; Schmidt et al., 2025).

## 3 Results

The results show the diverse experiences of midwives facing maternal deaths and emphasizing both personal and systemic reactions to these events, professional and emotional challenges, which are related to the multidimensionality of confronting maternal deaths. From the ten studies reviewed, three main themes were identified: *psychological impacts of maternal deaths on midwives, coping strategies, and institutional support* were identified following the presentation of results. The analysis indicates that aforementioned themes collectively elucidate a multifaceted interaction between psychological distress, adaptive coping mechanisms, and the systemic determinants impacting the well-being of midwives across the study period.

### 3.1 Psychological impacts of maternal deaths on midwives

The analysis of various studies indicates that maternal mortality events exert significant psychological and emotional impacts on midwives (Nkoane and Makhubela-Nkondo, 2024; Alemu et al., 2024; Guzzon et al., 2024). The data reveal that participants frequently reported experiencing emotions such as guilt, self-recrimination, sadness, anxiety, and symptoms of depression, even in scenarios where there was no direct culpability on their part. These emotional responses are indicative of an internalized sense of professional responsibility and moral distress linked to the demise of a patient under their care. The chronic exposure to such traumatic occurrences has been associated with emotional exhaustion, compassion fatigue, and burnout, particularly in environments characterized by elevated maternal mortality rates. This body of evidence underscores that maternal deaths extend beyond being mere professional obstacles, representing profound personal and psychological traumas that adversely affect the well-being and clinical efficacy of midwives.

### 3.2 Coping strategies

In addressing the psychological distress resultant from maternal mortality incidents, midwives employed a diverse array of coping mechanisms (Dartey et al., 2019b; Morton et al., 2021). Predominantly, peer support and informal debriefing sessions were reported as the primary strategies, offering environments conducive to shared reflection, emotional catharsis, and reciprocal understanding. Certain midwives sought professional counseling and mental health services when accessible; nonetheless, the availability and quality of these services exhibited significant variability across different settings. Furthermore, religious and spiritual practices were pivotal, providing solace and frameworks for meaning making, especially in culturally and spiritually inclined communities. The reliance on self-initiated and peer-based coping strategies underscores the tendency of midwives to navigate the deficit of structured institutional support by depending on interpersonal and spiritual resources to maintain their emotional fortitude.

### 3.3 Institutional support

The studies reviewed exhibit a notable heterogeneity in institutional support structures (Sheehy et al., 2025). Some healthcare facilities provide structured debriefing sessions, counseling services, and access to mental health professionals, thereby reflecting an organizational recognition of the psychological burden associated with maternal fatalities. In contrast, other institutions lack any formalized support mechanisms, resulting in midwives having to independently manage their grief and stress. This disparity highlights substantial deficiencies in institutional responses to occupational trauma, emphasizing the necessity for standardized psychosocial support frameworks and policies to protect the mental health and professional efficacy of midwives.

Emerging trends across studies highlighted the main outcome of this study identified three key trends: midwives frequently experience internalized psychological distress after maternal fatalities, coping strategies are mainly informal and peer-based, and institutional support is often inconsistent. These findings stress the importance of addressing midwives’ psychosocial needs to ensure quality maternal healthcare and workforce sustainability.

## 4 Discussion

This integrative review explored a comprehensive review that examines the psychological well-being of midwives who have experienced maternal deaths in their professional capacity. By analyzing research conducted between 2015 and 2023. The review provides insights into how these events emotionally affect midwives and evaluates the effectiveness of coping strategies and institutional support systems. The passage also indicates that the results of this analysis are discussed in detail, including their practical implications and suggestions for future research directions.

### 4.1 Psychological impact of maternal deaths on midwives

The review discovered that midwives frequently endure considerable psychological distress following the death of a patient, a reality that often goes unacknowledged. These findings are consistent with the previous studies that reported that emotional upheaval is characterized by deep-seated feelings of grief, guilt, sadness, and sometimes a profound sense of helplessness and inadequacy (Sökmen and Koç, 2024). Many midwives internalize these feelings, attributing blame to themselves even when circumstances were uncontrollable. Such emotional responses can intensify over time, leading to more serious mental health issues such as burnout, anxiety, and depression (Kartsonaki et al., 2023). This psychological burden is particularly noticeable among less experienced midwives or those without sufficient institutional or emotional support, hindering their ability to effectively process or manage distress (Hastings-Tolsma et al., 2021). In wealthier nations, maternal deaths occur infrequently due to superior healthcare infrastructure and support systems (Bingham et al., 2023). However, midwives in low- and middle-income countries often contend with significantly different conditions. Studies indicate that maternal mortality rates are substantially higher, and midwives frequently face considerable challenges like insufficient staffing, limited access to essential medical supplies, and excessive patient loads. These factors subject midwives to constant pressure, heightening their susceptibility to psychological stress and emotional exhaustion (Bingham et al., 2023). Moreover, the repercussions of maternal death are not confined to single events. Midwives might experience secondary or vicarious trauma, where the emotional burden of repeated exposure to traumatic incidents compromises their ability to deliver compassionate care to other patients (Mendelson, 2024). This cumulative trauma can impair their professional capabilities and reduce their job satisfaction. Continuous exposure to grief and loss in a high-pressure work environment amplifies the emotional burden and may have lasting psychological ramifications if not properly addressed (Uddin et al., 2022). Thus, the psychological repercussions of maternal deaths on midwives are significant and nuanced, necessitating enhanced institutional awareness, targeted mental health resources, and coping strategies to protect their well-being and sustain their critical role in maternal healthcare.

### 4.2 Coping strategies

Research indicates that midwives utilize various strategies to handle the psychological toll of maternal deaths. Emotional coping techniques are notably prevalent, with peer support and debriefing sessions serving as vital avenues for emotional expression and comfort. These gatherings often involve discussions with colleagues regarding the distressing incidents, assisting midwives in processing their emotions and reducing feelings of isolation. Beyond peer support, midwives often depend on family members or mental health professionals to help them manage the emotional repercussions of maternal fatalities (Maben et al., 2024; Marshman et al., 2023). Professional counseling and mental health services are also key coping tools, though their accessibility and sufficiency differ notably across healthcare environments. In resource-constrained settings, midwives frequently encounter significant obstacles in accessing these services, which can hinder their recovery from distressing events. In certain cultural contexts, religious or spiritual practices offer significant solace. Activities such as prayer, meditation, or other spiritual practices have been effective for some midwives in finding inner peace and resilience during challenging circumstances (Marshman et al., 2023; Schafer et al., 2024). Furthermore, strategies that enhance resilience, like self-care routines, organized peer support networks, and engaging in professional debriefings, are pivotal in mitigating psychological distress (Marshman et al., 2023; Mira et al., 2025). These practices support emotional management and assist midwives in preserving their mental health over time. Despite the presence of these strategies, many midwives indicate that these tools are often insufficient for fully addressing the profound psychological and emotional challenges following maternal deaths. Some midwives point out that the lack of formal psychological support systems in their work environments hinders their ability to effectively process trauma and achieve a healthy recovery (Urbańska et al., 2025; Parker et al., 2025).

### 4.3 Institutional support systems

The review highlighted the insufficient and irregular institutional support mechanisms available for midwives after experiences of maternal deaths. Several studies point out that structured approaches like regular debriefing sessions, psychological counseling, and peer support programs are either missing or not adequately utilized, especially in low-resource environments with limited healthcare facilities (Morton et al., 2021; Sheehy et al., 2025). These results align with previous research that indicated that numerous healthcare facilities encounter systemic constraints, including but not limited to staffing shortages, excessive patient-to-provider ratios, and restricted financial resources, which consequently detract from prioritizing the psychosocial welfare of healthcare personnel. This situation often results in midwives being compelled to address their emotional distress autonomously, thereby exacerbating their psychological susceptibility within inherently stressful clinical settings.

A key systemic driver of distress identified across multiple studies is the organizational culture prevalent within healthcare institutions. In various settings, midwives have reported encountering stigma and the apprehension of professional judgment when attempting to access psychological support (Argaheni et al., 2024; Kuforiji et al., 2023). The prevailing perception that emotional distress equates to weakness or professional inadequacy serves as a deterrent to the open discussion of mental health concerns. As a result, midwives frequently internalize their distress, suppress their emotional responses, and continue their professional duties while experiencing unresolved psychological strain. This institutional silence fosters a persistent cycle of burnout, compassion fatigue, and moral injury. Furthermore, organizational responses to maternal deaths are predominantly procedural or punitive, focusing on clinical audits, administrative inquiries, and risk management, rather than prioritizing the well-being of the staff (Stabnick et al., 2022). Such approaches fail to consider the emotional impact of maternal deaths on frontline midwives, leaving their psychological needs largely unaddressed despite the significant and enduring nature of the trauma (Eagen-Torkko et al., 2021).

From a systemic viewpoint, these findings highlight the lack of a comprehensive institutional structure for emotional recovery. The absence of proactive leadership involvement in establishing mental health policies and culturally aware support mechanisms indicates a larger deficiency in healthcare governance. Without cohesive psychosocial strategies, institutions risk maintaining environments where the mental health of midwives is secondary to clinical outcomes. Consequently, the review advocates for a paradigm shift—from reactive, outcome-focused management to preventive, person-centered organizational care that incorporates psychological support as a standard element of post-incident practice.

A comprehensive critical assessment of the diversity and methodological restrictions of the studies examined reveals that, although they collectively provide significant insights, there are several methodological limitations that need to be recognized. A significant number of these studies depended on self-reported data, which may be subject to recall bias, emotional sensitivity, or social desirability bias, given the stigma associated with mental health issues in professional environments. The diversity in study designs, which includes qualitative interviews, focus groups, and small-scale cross-sectional surveys, imposes restrictions on the comparability and generalizability of the findings. Additionally, the majority of the research was conducted in specific geographic or cultural settings, predominantly within low- and middle-income countries where healthcare resources are limited. Therefore, cultural norms, religious beliefs, and institutional hierarchies that influence midwives’ coping mechanisms may not be entirely applicable to other contexts.

## 5 Recommendations for future research and practice

This review emphasizes the urgent need for strategies to support midwives’ psychological well-being after maternal deaths, noting gaps in institutional support, reliance on informal coping, and limited research on effective interventions drawn from the reviewed studies. Based on the review findings, in this study, the authors recommended that subsequent research should prioritize examining the lasting psychological impacts on midwives following maternal fatalities, particularly focusing on how repeated traumatic experiences influence their emotional state across diverse healthcare contexts. It is important to assess coping mechanisms and initiatives aimed at enhancing resilience, including peer support, debriefings, and mental health services. Further research should consider the incorporation of personalized psychological support within workplace settings and evaluate the creation of institutional structures that support the mental health of midwives, especially in resource-constrained environments. The authors further recommended that leadership in-service training should equip healthcare leaders with the skills to detect and manage midwives’ distress. Finally, the adoption of trauma-informed policies and enduring mental health services is crucial to provide consistent support throughout midwives’ professional lives. The recommendations highlight the need for a holistic approach to support midwives, combining evidence-based research, institutional policies, and culturally sensitive mental health practices, to improve midwives’ psychological well-being and enhance maternal care quality and empathy.

The implication is that improving midwives’ psychological well-being requires systemic interventions beyond personal coping strategies. Healthcare institutions should embed evidence-based mental health support in their policies to prioritize emotional care for midwives and patient safety. Culturally sensitive strategies ensure mental health interventions are relevant and accessible in diverse settings. Strengthening institutional commitment to psychological support enhances midwives’ resilience and improves maternal care quality, empathy, and outcomes.

## 6 Conclusion

This comprehensive review elucidates that maternal mortality events exert a significant and lasting psychological impact on midwifery professionals, which detrimentally influences both their psychological well-being and professional efficacy. The studies analyzed consistently indicate that midwives experience considerable emotional distress, characterized by feelings of guilt, anxiety, and burnout, exacerbated by insufficient institutional support mechanisms. While individual resilience and peer-based coping stratagems provide some mitigation, the lack of structured organizational supports—such as debriefing sessions, counseling services, and a supportive workplace culture—constitutes a notable deficiency, especially in resource-limited environments. To ameliorate these issues, healthcare institutions must prioritize the implementation of robust psychological support systems for midwives. Such systems should encompass routine debriefing, readily available counseling services, and leadership practices that endorse emotional care as a normative aspect of the professional environment. Furthermore, integrating psychological resilience and self-care training within midwifery curricula would better prepare practitioners to manage the emotional challenges inherent in their profession. Collectively, these interventions have the potential to enhance both the mental health of midwifery practitioners and the overall quality of maternal care provided.

## 7 Limitations of the review

The limitations of this review include the lack of longitudinal and intervention-based studies and the lack of high-quality randomized controlled trials, which should be considered in interpreting the results. Additionally, there was a lack of diversity in the types of coping mechanisms discussed across different cultural settings. It notes a need for more theory-driven, multi-site investigations to enhance evidence-based recommendations. Although there are differences in methods, research consistently indicates that institutional neglect and systemic failures play a significant role in causing emotional distress among midwives. Further research should address these gaps, exploring the impact of maternal deaths on midwives in various cultural and healthcare contexts.
